# Social Media and Science

**DOI:** 10.21470/1678-9741-2020-0384

**Published:** 2020

**Authors:** Marcelo Luiz Peixoto Sobral

**Affiliations:** 1 Departamento de Cirurgia Cardiovascular, Hospital Beneficência Portuguesa de São Paulo, São Paulo, Brazil.

The importance of social media (SM) in the practice of medicine has been increasingly emphasized, and the main argument is that technology should be used in favor of science. Of course, there are some limits and responsibilities to be followed. Thinking carefully, when a researcher or any other professional carries out a scientific paper, it aims at publishing to disseminate this knowledge to the scientific community, students, patients, journalists, industry and even project financing institutions can benefit from it^[1]^. And why not make the most of it?

Therefore, we must define a few basic concepts of the use of SM to provide a better perspective on the dissemination of knowledge to the clinical and academic areas. For example, Twitter has an emerging role in the dissemination of health information and used by one of the leading cardiovascular journals and professional organizations, such as the American College of Cardiology, is able to disseminate information about cardiovascular disease and health education quickly, efficiently, and on a worldwide scale. To exemplify this expansive social media feature, the *New England Journal of Medicine* had 1 Tweet re-tweeted by a user with 560,000 followers^[2]^.

In addition, Ladeira-Lopez in 2020 concluded, after a preliminary analysis of three-quarters of the randomized articles of the European Society of Cardiology Journals Randomized Study, that a social media strategy to promote cardiovascular medicine papers on Twitter seems to be associated with increased online visibility and greater number of citations, showing an association between an active promotion of papers on Twitter and their online visibility by Altmetric* system and scientific impact on citations^[3]^.

Another important tool is the hashtag, which is a type of metadata tag used in SM, and that makes it possible to easily find messages with a specific theme or content. Of the 467 hashtags used to denote content of interest to the cardiovascular community, the two most used were: #CardioTwitter and #Stroke, in which they were used for a wide and varied discussion of topics^[4]^.

Thus, the power of SM to communicate openly, with wide access around the world and at a faster speed than ever before, makes it a formidable strength^[5]^. Given that condition, SM has been transforming the medical community in a short time and irreversibly, generating impact and influence on important strategic decisions. Rather than resisting, we must embrace this idea. However, future research will be necessary to understand the synergies between SM and evidence-based practice, as well as to develop institutional policies that benefit patients and healthcare professionals.

Do not be afraid to take a step forward!

## Footnotes

(*) Altmetric (www.altmetric.com) is a system that tracks the attention that research outputs, such as scholarly articles and datasets, receive online. It pulls data from: social media (Twitter and Facebook); traditional media (mainstream and specific field); non-English language titles are covered; blogs (major organizations and individual researchers); online reference managers (Mendeley and CiteULike).
